# Draft Genome Sequences of *Corynebacterium kroppenstedtii* CNM633/14 and CNM632/14, Multidrug-Resistant and Antibiotic-Sensitive Isolates from Nodules of Granulomatous Mastitis Patients

**DOI:** 10.1128/genomeA.00525-15

**Published:** 2015-05-21

**Authors:** Maria Isabel Fernández-Natal, Francisco Soriano, Jaime Ariza-Miguel, Teresa Marrodan-Ciordia, Alberto Acedo, Marta Hernandez, Andreas Tauch, David Rodríguez-Lázaro

**Affiliations:** a Department of Clinical Microbiology, Complejo Asistencial Universitario de León, Spain; b Institute of Biomedicine (IBIOMED), León, Spain; c Public Health, School of Physiotherapy ONCE, Madrid, Spain; d Instituto Tecnológico Agrario de Castilla y León (Itacyl), Valladolid, Spain; e AC-Gen Reading Life, Valladolid, Spain; f Institut für Genomforschung und Systembiologie, CeBiTec, Universität Bielefeld, Bielefeld, Germany; g Microbiology Section, Department of Biotechnology and Food Science, Faculty of Science, University of Burgos, Burgos, Spain

## Abstract

*Corynebacterium kroppenstedtii* has been associated with infections of the female breast. Genome sequencing of two strains revealed a specific genomic island in the multidrug-resistant isolate CNM633/14 with similarity to the R plasmid pJA144188 of *Corynebacterium resistens* DSM 45100, being indicative of the horizontal transfer of antibiotic resistance genes to *C. kroppenstedtii*.

## GENOME ANNOUNCEMENT

*Corynebacterium kroppenstedtii* is a lipophilic corynebacterial species lacking the characteristic mycolic acids of the cell envelope (1, 2). The lack of corynemycolic acids and the lipophilic lifestyle of *C. kroppenstedtii* are caused by gene loss, including a condensase gene cluster and a mycolate reductase gene, both involved in mycolic acid biosynthesis, and a microbial type I fatty acid synthase gene, resulting in a fatty acid auxotrophy (3). Since the first isolation of *C. kroppenstedtii* from sputum of an 82-year-old woman with chest infection (1), it has been obtained mainly from women’s breasts and has been associated with granulomatous mastitis (4–10) and breast abscesses (2, 11, 12). To attain genetic knowledge of this pathogen, we sequenced the genomes of two clinical isolates from nodules of granulomatous mastitis patients. The antibiotic-sensitive strain *C. kroppenstedtii* CNM632/14 (also known as strain ITA205) was isolated from a 38-year-old woman with a nodule in her upper lateral right breast, successfully treated with cefuroxime and corticosteroids. The multidrug-resistant strain *C. kroppenstedtii* CNM633/14 (also known as strain ITA205) was isolated from a 45-year-old woman suffering granulomatous mastitis, initially treated unsuccessfully with ciprofloxacin and corticosteroids and finally with cefuroxime and corticosteroids.

Both *C. kroppenstedtii* isolates were routinely grown at 37°C on blood agar. Genomic DNA was purified by using the ChargeSwitch genomic DNA (gDNA) mini bacteria kit (Invitrogen). Ion Torrent PGM data were generated from two different libraries constructed from 200 ng of DNA quantified with the Qubit Fluorometer 2.0 (Life Technologies). Fragment libraries were constructed with the Ion Xpress Plus fragment library kit comprising the Ion Shear DNA fragmentation chemistry. Barcode and adaptor ligation were performed using the Ion Xpress barcode adapters 1-16 kit. Quantitation and size distribution of fragments were analyzed on an Agilent Bioanalyzer with the high-sensitivity DNA kit. Template preparation and emulsion PCR were performed using the Ion PGM template OT2 200 kit. The product was quantitated with the Ion Sphere quality control kit on the Qubit Fluorometer 2.0. The Ion OneTouch ES was used for enrichment of the Ion Sphere particle template products. Finally, the samples were loaded into an Ion 316 Chip v2 and sequenced using the Ion PGM sequencing 200 kit v2. Reads were collected by the Torrent Suite software v4.0, which also sorts the data according to the barcodes used. The MIRA program (version 3.4.0) (http://www.chevreux.org) was used for *de novo* assembly of the two genomes.

Both genome sequences were annotated using the RAST genome annotation server (13). The two draft genome sequences revealed a high grade of similarity (99.97%). BLAST was used for the search for antibiotic resistance genes in the Antibiotic Resistance Genes database (14) and in published genome information of *Corynebacterium* strains. The antibiotic resistance determinants of the multidrug-resistant isolate *C. kroppenstedtii* CNM633/14 were allocated to a specific genomic island with similarity to the R plasmid pJA144188 of *C. resistens*, including the resistance genes *erm*(X), *tet*(W), *cmx*, *aphA1-IAB*, *strA*, *strB*, and *sul1* (15).

### Nucleotide sequence accession numbers.

The whole-genome shotgun projects for the two *C. kroppenstedtii* strains have been deposited in the GenBank database under the accession numbers JYCR00000000 (CNM633/14) and JYDD00000000 (CNM632/14).
